# Dementia-related agitation: a review of non-pharmacological interventions and analysis of risks and benefits of pharmacotherapy

**DOI:** 10.1038/tp.2017.199

**Published:** 2017-10-31

**Authors:** E O Ijaopo

**Affiliations:** 1 Department of Medicine, Royal Wolverhampton NHS Trust, New Cross Hospital, Wolverhampton, West Midlands, UK

## Abstract

Unsurprisingly, the subject of dementia has been a rising matter of public health concerns as people now live longer. World Alzheimer Report 2015, estimate that about 46.8 million people worldwide have dementia. These numbers are projected to almost double every 20 years, reaching 74.7 million in 2030 and 131.5 million in 2050. The modality for treating agitation and other behavioral symptoms in dementia patients has been a challenge. Many years on, there has been no FDA-approved pharmacotherapy in treating dementia-related agitation. This review discusses the current knowledge of non-pharmacological interventions, and analyzes the risks and benefits of pharmacotherapy in the management of dementia-related agitation, as well as providing an anecdotal of the author's clinical experience. This article aims to provide opportunity for increase awareness for clinicians, particularly those with no specialty training in geriatrics medicine but see dementia patients with agitation and other behavioral symptoms from time to time. Likewise, it hopefully will benefit the readers of medical journals to update their existing knowledge on matters relating to the management of dementia-related agitation.

## Introduction

Because of increased longevity, the number of people being diagnosed with dementia is on the rise. In the year 2015 alone, an estimated 9.9 million new cases of dementia was expected to be diagnosed worldwide, equivalent to one new case of dementia in every 3.2 s.^1^ World Alzheimer Report in 2015 estimates about 46.8 million people worldwide have dementia. These numbers are projected to almost double every 20 years, reaching 74.7 million in 2030 and 131.5 million in 2050. In the United States of America, the Alzheimer's Association claims over 5 million of Americans have dementia, and by the year 2050, it is estimated that up to 16 million Americans will have dementia.^2^

The 2015 global societal economic cost of dementia was estimated at US$818bn, and this is expected to rise to a trillion dollars by the year 2018.^1^ In the United States, in 2010, the yearly monetary cost per person with dementia varied from $41 689 to $56 290.^3^ The American Alzheimer's Association claim the monetary costs alone attributable to dementia were $172 billion.^4^

Unfortunately, during the course of their disease, over 90% of dementia patients will suffer from psychiatric or behavioral problems commonly referred to as neuropsychiatric symptoms.^5, 6^ Neuropsychiatric symptoms in dementia patients include agitation, delusions, hallucinations, dysphoria, anxiety, aggression, euphoria, disinhibition, irritability/lability, apathy and aberrant motor activity that include wandering.^7, 8^ Agitation, being one of the most commonly observed neuropsychiatric symptoms, is reported to be found in up to 70% of dementia patients.^9, 10, 11^ A longitudinal observational study in Dutch nursing homes, where 372 residents with dementia were followed up for 3.5 years (2007–2011) to assess the prevalence and course of symptoms exhibited during dementia illness from the time of admission into care facility to the time of death, found agitation to be the most common prevalent symptom, accounting for up to 71%.^12^ Agitation can be described as restless behavior, or improper physical and verbal actions that may cause trouble for family members, caregivers and other service users. Quite commonly, it is associated with caregiver burden that results in the caregivers getting burnout.^13^ Likewise, agitation in one resident may trigger agitation in the other residents.^14^

Recommended management for dementia-related agitation and other behavioral symptoms involve non-pharmacological interventions and pharmacotherapy. The American (including the American Associations for Geriatric Psychiatry), British and Canadian Geriatrics Societies recommend non-pharmacological interventions as the first-choice approach in managing dementia-related agitation. However, in emergency situations when non-pharmacological approach is not feasible due to an imminent danger to patient's safety, drug therapy is recommended as a first-line treatment intervention. However, many years on, there is still no Food and Drug Administration (FDA)-pharmacotherapy in treating dementia-related agitation. This review discusses the current knowledge of non-pharmacological interventions and analyses the risks and benefits associated with pharmacotherapy in the management of dementia-related agitation, as well as providing an anecdotal of the author's clinical experience.

## Methods

A computer search of databases including PubMed, MEDLINE, CINAHL, PsycInfo and EMBASE were carried out to identify studies in peer-reviewed journals published within the last dozen years, from January, 2005 to March, 2017. Search terms used were: treatment for dementia agitation, drugs treatment/pharmacotherapy and non-pharmacological treatment/interventions, management of neuropsychiatric behavior in dementia (because some studies included agitation as part of the neuropsychiatric behavior). Systematic reviews, meta-analyses and randomized trials (of pharmacologic and non-pharmacologic treatments), were included for review. Likewise, practical guidelines for dementia-related agitation were review. A manual search of additional references was also done among the references found in the databases. Clinical trials.gov was also reviewed for completed and ongoing trials related to treatment of agitation in dementia. Only studies conducted in English language were preferentially reviewed. Data synthesis and recommendations from this review came from available evidence obtained from the studies reviewed and the anecdotal clinical experience of the author.

## Non-pharmacological interventions

The growing concerns over the efficacy and side effects of pharmacotherapy have resulted in the emphasis of non-pharmacological interventions as the first line treatment approach.

NICE UK in agreement with the American and the Canadian Association for Geriatric Psychiatry, as well as the European Association of Geriatric Psychiatry (EAGP) recommends the initial treatment approach for people who have agitation in all types and severities of dementia to be the non-pharmacological interventions.^33^

This approach includes simple and complex interventions which are most often combined and provided to patients in a person-centered care approach. Examples of these interventions include multisensory stimulation, aromatherapy, music therapy, cognitive behavioral therapy, animal-assisted therapy, electroconvulsive therapy (ECT) and physical exercises. These non-pharmacological interventions are increasingly being recognized globally as the crucial parts of the comprehensive dementia care due to its fewer risks compare to the drugs treatment. Unenthusiastically however, available evidence still shows that effective non-pharmacological interventions have not been widely embraced by the real-world clinical practice and standard of care.^10, 18, 34, 35, 36^ Kales *et al.* recommend a non-pharmacologic strategy called DICE approach to be used as a basis for integrating non-pharmacologic and pharmacologic interventions in the real-world clinical settings.^35^ The DICE approach which comprises Describe, Investigate, Create and Evaluate, is essentially patient/caregiver-centered approach that describes sequential steps for thorough assessments to help manage neuropsychiatric symptoms of dementia. Kales *et al.* claim DICE model will provide clinicians with well-planned efficient treatment approach when integrated into clinical practice. Another model called Targeted Interdisciplinary Model for Evaluation and treatment of neuropsychiatric symptoms (TIME) was described by Lichtwarck *et al.* as a multicomponent intervention program frequently employed by physicians and nursing home staff (in Norway) for assessing and treating behavioral and psychological symptoms of dementia or other complex disorders.^37^ The TIME model consists of three interconnected phases of registration and assessment phase; a guided reflection phase; and an action and evaluation phase. These phases were extracted from the concepts of cognitive behavioral therapy and are to be tailored to individual patient. In 2016, Lichtwarck *et al.*, published a study protocol on the proposed 3-month TIME trial to take place in 30 nursing homes (in Norway) involving 168 residents with high degree of dementia-related agitation, so as to evaluate the effectiveness-implementation of TIME model and its implementation process by staffs and at organization levels. The authors believe the result of TIME intervention model could become an evidence-based model that will improve assessment and treatment of agitation and other neuropsychiatry symptoms in dementia patients.

Within the last decade alone, numerous studies (Abraha *et al.*;^19^ Cohen-Mansfield *et al.*;^38^ Rapp *et al.*;^39^ Blythe *et al.*;^40^ Cooke *et al.*;^41^ Jutkowitz *et al.*;^22^ Lin *et al.*;^42^ van der Ploeg *et al.*;^43^ Van Vracem *et al.*^44^) have been published describing the effectiveness of the non-pharmacologic interventions in the prevention and treatment of dementia-related agitation (Tables 1 and 2).

Hawranik *et al.*^15^ and Woods *et al.*^16^ describe the potential usefulness of therapeutic touch as playing significant positive role in managing dementia-related agitation and other behavioral symptoms. The combination of therapeutic touch using acupressure and psychosocial interventions using Montessori activities also recorded positive findings toward decreasing agitation in residents with dementia.^45^ Listening to familiar music is shown to demonstrate significant effectiveness in reducing agitation and other related behavioral and psychiatric symptoms in patients with moderate to severe dementia.^46, 47^ Also, proven to be of significant clinical benefit in reducing agitation are the tailored activities program channeled to the capability of individual dementia patients.^48^ Gitlin *et al.*^48^ add that the tailored activities training provided to families/caregivers also result in beneficial effects which reduce caregiver burden and improved skills among other benefits.

Conventionally, it is long believed that lavender has some of therapeutic and curative properties. Neurophysiological and animal studies according to Koulivand *et al.*^49^ report that lavender oil may have some effectiveness in the treating several neurological disorders due to its anxiolytic, sedative and neuroprotective properties. During that interval, O’Connor *et al.*^17^ undertook a randomized single-blind cross-over trial of dermally-applied, physiologically active, high purity 30% lavender oil versus an inactive control oil on 64 nursing home residents with frequent physically agitated behaviors. They found that despite its sedative and anxiolytic properties, lavender oil showed no significant advantage over control oil and demonstrated no evidence in reducing the agitation behavior in dementia patients.

Further, in 2014, Livingston *et al.*^18^ conducted systematic review of 160 studies that investigated non-pharmacological interventions for agitation in dementia people over 50 years of age in care facility settings. They reported that engaging agitated dementia patients on various activities including music therapy and sensory interventions (massage, therapeutic touch and multisensory stimulation) may help to reduce mild-to-moderate agitation in the immediate term, but, lacked significant long term benefits, and no beneficial effects on severe agitation symptoms. However, caregivers trained and supervised in communication or person-centered skills demonstrated immediate improvement on severe agitation with effects lasting up to 6 months. In contrary, light therapy (of 30–60 min daily bright-light exposure) and aromatherapy interventions reported no evidence of benefit in reducing agitation.

Meanwhile, as agitation in dementia patients may be due to other causes, focusing to alleviate pain and sleep disorders problems, and discontinuation of inappropriate medications may markedly help the management of behavioral symptoms related to dementia.^50^ Likewise, dealing with hypothesized unmet needs of dementia patients play remarkable role in managing agitation-related symptoms in dementia.^38^ The latter point was further buttressed by Jakobson *et al.*^10^ who demonstrated that patient specific intervention programs that aim to address unmet needs have proven evidence in reducing agitation in dementia patients.

Abraha *et al.*^19^ present an overview of nonpharmacological interventions for behavioral and psychological symptoms in dementia through systematic overview of 38 systematic reviews and 142 primary studies comprising different categories of nonpharmacological interventions.^19^ They studied various sensory stimulation interventions (acupuncture, aromatherapy, massage therapy, light therapy, sensory garden intervention, cognitive stimulation, music/singing and dance therapy, snoezelen and transcutaneous electrical nerve stimulation (TENS) therapy); with cognitive/emotion-oriented interventions; behavioral management technique; and other interventions including exercise therapy, pet-therapy and special care unit. Abraha *et al.*^19^ found that music therapy is the only effective nonpharmacological sensory intervention that reduces agitation in dementia patients. This finding is corroborated by other previous studies (Ridder *et al.*;^14^ Sung and Chang;^47^ Ray and Mittelman^51^), which demonstrated significant beneficial effects of music therapy in reducing symptoms of agitation in dementia patients. Meanwhile, in another randomized trial by Vink *et al.*^21^ comparing the effects of music therapy with general day activities on agitation in 77 dementia persons resident in nursing home, claimed that both music therapy and recreational activities only resulted in short-term decrease in agitation, and that music therapy did not show any additional beneficial effects over general activities.

Further analysis by Abraha *et al.*^19^ claims that some behavioral management techniques directed at enhancing staff communication skills, formal caregiver training and dementia mapping were effective at reducing agitation. But, interventions such as exercise and pet therapy failed to show any beneficial effects on the behavioral and psychological symptoms of dementia patients. Meanwhile, quite oppositely, a systematic review and meta-analysis of nineteen studies by Jutkowitz *et al.*^22^ to evaluate the efficacy of selected nonpharmacological care-delivery interventions including staff training, care-delivery models and environment changes in reducing agitation and aggression in care home dementia residents found insufficient evidence to prove that the behavioral management techniques are any more effective than the usual care in improving agitation and aggression in dementia patients.

When assessing the safety of ECT, Acharya *et al.*^9^ analyzed 23 subjects who had 12 ECT courses to treat their symptoms of agitation and aggression. They report that the use of antipsychotic medications for agitation and aggression symptoms was significantly reduced during the period of the ECT treatment. Acharya *et al.*^9^ suggest that ECT may be considered a suitable, safe and effective treatment for some patients with dementia whose behavioral disturbances are unresponsive to the standard non-pharmacological interventions and pharmacotherapy, or are not able to tolerate the latter.

In another experimental study, Yang *et al.*^11^ assess the efficacy of aroma-acupressure and aromatherapy in the treatment of dementia-related agitation. Although agitation improved in both groups, aroma-acupressure showed a better effect than aromatherapy in reducing agitation in dementia patients. They recommend future studies are necessary to further explore the benefits of aroma-acupressure and aromatherapy in treating agitation in dementia patients.

Several further studies have discussed various positive findings that promote the safety and efficacy of non-pharmacological approaches at improving dementia-related agitations in care facilities.^8, 36, 52^ Although, a recent review by Abraha *et al.*^20^ that analyzes three studies of differing design, involving 144 dementia persons, assessing whether simulated presence therapy intervention using video or audiotape recordings of family members played to dementia person, helps in reducing agitation and distressed symptoms. They concluded there to be inadequate evidence available to believe simulated presence therapy intervention has significant efficacy in treating behavioral and psychiatric symptoms of dementia patients.

Despite the general recommendation of non-pharmacological approach as the first-line choice in treating dementia-related agitation and the available evidences supporting some of them, a survey study conducted by Cohen-Mansfield *et al.*^53^ in Israeli nursing homes, to examine physicians' actual practice in treating dementia-related agitation and to check their familiarity with use of non-pharmacological interventions showed that 92.5% of physicians prescribed psychotropic medications for treating agitation. Quite astonishingly, the 67 physicians studied show low-familiarity levels with the use of non-pharmacological interventions. More so, as can be predicted, physicians with non-specialty in geriatrics were noted to have higher levels of unfamiliarity with non-pharmacological interventions.

With so much being said, the non-pharmacologic interventions are not without challenges. They are sometimes more difficult to carry out and could take longer time to be effective. They are also best tailored to individual clinical needs. Little wonder, their uptake as the preferred treatments for dementia-related agitation and neuropsychiatric symptoms remains limited in the real-world clinical settings. More so, even as most evidence of non-pharmacologic interventions available from literatures show good efficacy in the early and mid-stages of dementia, whether these interventions are effective in the later stages of dementia remains unclear.^54^

Finally, it is important to note that agitation may occur as an end-of-life symptom in people with advanced dementia, Eapcnet.eu^55^ discusses the palliative care approach to managing behavioral and psychological symptoms of dementia (BPSD) which may include agitation behavior that challenges caregivers, or be distressing for the patient. In these situations, they recommend getting the opinions of geriatricians or dementia care/palliative care specialists. van der Steen *et al.*^56^ further elaborated on the European Association for Palliative Care (EAPC) recommendations and provide a framework guidance for clinical practice, research and policy. To further buttress the need for palliative care approach in managing agitation symptoms in advanced dementia, Hendriks *et al.*^57^, reviewed the qualities of life of 330 nursing home residents with dementia who were in their last week of life and found that 35% of them had agitation symptoms.

## Risks and benefits of pharmacological interventions

Quite often, it is not uncommon that physicians use pharmacotherapy approach for treating dementia-related agitation and other neuropsychiatric symptoms (NPS). Several drugs including neuroleptics, anti-depressants, sedatives/hypnotics and anxiolytics among others are frequently used. While there is still no FDA-approved drug treatment for dementia-related agitation, they have issued several black box warnings regarding increased risk of stroke and mortality associated with the use of some drugs (particularly antipsychotics) in the elderly patients with dementia.^31, 58^

Ballard *et al.*^5^ conducted a randomized trial across five areas in Great Britain, to examine dementia patients who were started on long-term (at least 3 months) neuroleptic agents for neuropsychiatric symptoms control. They assess whether neuroleptics affect cognitive function and other outcomes, and determine whether discontinuing neuroleptics was related to an exacerbation of neuropsychiatric symptoms. Ballard *et al.*^5^ report that withdrawal of neuroleptics in most patients with Alzheimer's disease led to measurable improvement of cognitive and functional status, and no significant harmful effect. A year later, in 2009, Rappaport *et al.*^23^ did a randomized, double-blind, placebo-controlled tolerability study of intramuscular aripiprazole on 129 acutely agitated patients with Alzheimer's, vascular, or mixed dementia across 16 centers in the USA. Moderate-to severe acute exacerbations of agitated behavior was defined using Positive and Negative Syndrome Scale-Excited Component (PEC) scores, and they performed efficacy analyses for the PEC, Agitation–Calmness Evaluation Scale (ACES), Clinical Global Impressions–Severity of Illness (CGI-S) and Clinical Global Impressions–Improvement (CGI-I) rating scales. The authors concluded that a total of 10 mg or 15 mg of intramuscular aripiprazole given in divided doses showed overall good tolerability and remarkable improvements in agitation associated with Alzheimer's, vascular, or mixed dementia. Similarly, another study by Seitz *et al.*^59^ on several atypical antipsychotics (risperidone, olanzapine and aripiprazole) showed demonstrable good evidence in managing neuropsychiatric symptoms of dementia patients. But, Seitz *et al.*^59^ recommend that safe and effective non-pharmacological interventions approach should be considered ahead of antipsychotics and other pharmacological treatments due to distressing adverse effects. Schneider *et al.*^60^ demonstrated that atypical antipsychotics are associated with an increased risk of death, with an odds ratio of 1.54. Likewise, observational study by Gill *et al.*^61^ found an increased risk of mortality with the use of the antipsychotics. There is also reported evidence of increased risk of major cerebrovascular events associated with antipsychotics use.^62^ Other common adverse effects which should also be monitored during treatment with atypical antipsychotics include increased falls and fall-related injuries such as hip fractures.^63^ Most often, these risks occur soon after initiating antipsychotics treatment, although chronic therapy is also argued to be associated with increased risks.^5, 54^

In a 12-week prospective study involving sixteen Alzheimer's disease (AD) patients with significant agitation by Cakir and Kulaksizoglu,^24^ it was believed that mirtazapine has some effectiveness in treating agitation related to AD. Mild side-effects such as sedation, headache, increased appetite and mild hypotension relating to mirtazapine were reported in the study. Comparably, Wang *et al.*^25^ study of 22 nursing home and community dwelling patients with probable Alzheimer's disease who had agitation and aggression randomized to an 8-week single-site study of placebo versus prazosin concluded that prazosin, compared with placebo, demonstrates remarkable improvement at reducing agitation and aggression in AD patients. They observed that prazosin was well tolerated by the participants and very rarely associated with sedation which is one of the worrying side effects of neuroleptics.

Donepezil and memantine are frequently used in mild-to-moderate and moderate-to-severe symptoms of AD respectively. Their roles as monotherapies in managing the BPSD within their licensed indications were compared in a systemic review and meta-analytic study by Lockhart *et al.*^26^ analyzed six randomized controlled monotherapy trials (four donepezil and two memantine) and findings showed that donepezil monotherapy within its licensed indication is found to be significantly more efficacious than placebo for alleviating BPSD. On the other hand, memantine monotherapy within its licensed indication failed to show any statistically significant advantage over placebo in managing BPSD. Fox *et al.*^27^ findings also buttressed the claim that memantine has no significant advantage over placebo in improving agitation in people with moderate-to-severe dementia. Exploring efficacy of memantine further, Herrmann *et al.,*^28^ organized a randomized, double-blind placebo-controlled trial on 369 patients with moderate-to-severe AD over 24 weeks period to assess the efficacy of memantine over placebo in reducing agitation and aggression-related symptoms. Several standardized tests scores (see Appendix) employed to measure the outcome showed no statistically significant benefit of memantine over placebo in reducing the symptoms of agitation and aggression in moderate-to-severe AD. Quite contrarily, Li *et al.,*^29^ claim memantine treatment has beneficial effects on neuropsychiatric symptoms of patients with moderate-to-severe form of behavioral variant frontotemporal dementia (bvFTD) after conducting a six-month, open-label, self-controlled clinical trial on 42 outpatients. The patients were treated with 20 mg memantine daily (as was the case with Herrmann *et al.*^28^) for six months’ duration. The standardized neuropsychiatric tests scores used to compare their baseline scores with endpoint scores after six months treatment reported much better improvements to memantine in patients with moderate-to-severe bvFTD, but found no beneficial effects of cognitive function and behavioral symptoms in patients with mild bvFTD. Obviously, the study report needs to be interpreted with care as the possibility of spontaneous symptoms resolution could upswing the data resulting in the statistically significant improvement in the moderate-to-severe bvFTD patients. Quite arguably as well, the lack of beneficial effects in patients with mild bvFTD may be related to compliance issues, particularly if the patients did not feel too unwell in themselves to justify the necessity for treatment compliance lasting six months period. More importantly, as claimed by the authors, the low power of the study also explains why extra caution must be used when applying the trial outcome to clinical practice.

In another study, a drug called mibampator (an AMPA receptor potentiator) was studied against placebo treatment in 132 AD patients with agitation and aggression who were randomized to 12 weeks of double-blind treatment.^30^ The trial findings show no evidence of advantage of mibampator over placebo in treating agitation and aggression in AD patients

Further, in 2014, Porsteinsson *et al.*^31^ conducted a 9-week double-blind randomized trial on 186 probable AD patients with agitation across 8 academic centers in Canada and United States. They found that among the patients with probable Alzheimer disease-related agitation who were receiving psychosocial intervention, the addition of citalopram compared with placebo leads to significantly reduced agitation. However, citalopram dosage may have to be limited to 30 mg per day due to worsening of cognition and worrying cardiac (QTc prolongation) adverse effects noted in patients. Porsteinsson *et al.* conclude that evidence available to assess the efficacy of citalopram at lower doses for treating agitation is insufficient. However, earlier in 2012, FDA^58^ updated their safety warnings that citalopram was associated the dose-dependent risk of QT prolongation which may cause Torsade's de Pointes, ventricular tachycardia and sudden death. They advised dose restriction with a maximum recommended dose of 20 mg per day for patients who are older than 60 years; have liver impairments; or taking other drugs (CYP2C19 inhibitor) that can increase blood levels of citalopram. Drye et al.^64^ study data reinforce the FDA warning against the use of higher doses of citalopram in people over 60 years, and report that Citalopram, at 30 mg per day, reduces agitation in patients with Alzheimer's disease but also causes concerning cognitive effects and QT prolongation.

Further study in 2016 by Ho *et al.*^65^ compared the relationship between (R) and (S)-citalopram enantiomer exposure and explored their therapeutic response on agitation behaviors in elderly AD population. Their analysis showed evidence that (S)-citalopram (escitalopram) is more efficacious than (R)-citalopram in the treatment of agitation in an elderly people with AD patients. In addition, exposure to (R)-citalopram revealed association of more adverse effects compared with (S)-citalopram exposure which established significant therapeutic benefit and a better choice than racemic (R) citalopram.

Meanwhile, in a published 10-week placebo-controlled randomized clinical trial, Cummings *et al.*^32^ study 220 probable AD patients with agitation to assess the efficacy, safety and tolerability of dextromethorphan-quinidine sulfate. The combination of dextromethorphan-quinidine treatment compared with placebo demonstrated clinically relevant efficacy for agitation management and was also found to be generally well-tolerated. While some adverse events including falls, diarrhea and urinary tract infections were reported with the dextromethorphan-quinidine treatment, there was however, no associated cognitive impairment, sedation or clinically significant QTc prolongation.

It is good to note that several ongoing clinical trials are focusing on novel drugs for treating agitation in dementia population. Among these is the clinical trial by Intra-Cellular Therapies, who are conducting a multicenter, randomized, double-blind, placebo-controlled Phase 3 clinical trial on ITI-007-201 to evaluate the efficacy, safety and tolerability of ITI-007 in patients with a clinical diagnosis of probable AD with clinically significant agitation.^66^ The study anticipates to enroll 360 subjects that will be randomly assigned to receive a one-month course of 9 mg per day of ITI-007 against placebo.^66^ Similarly, University of Sussex in United Kingdom is carrying out phase 3 clinical trial on mirtazapine or carbamazepine use for agitation in dementia (SYMBAD).^67^ The authors aim to assess whether Mirtazapine or Carbamazepine are more effective than placebo in treating agitation in people with dementia. The trial will assess the safety, clinical and cost effectiveness of each treatment over 12 weeks period and will follow the participants for up to one year.^67^

A phase 3, multicenter, randomized, double-blind, placebo-controlled study by Avanir Pharmaceuticals is also ongoing.^68^ The trial aims to assess the efficacy, safety and tolerability of AVP-786 (Deuterated [d6]-Dextromethorphan Hydrobromide [d6-DM]/Quinidine Sulfate [Q]) for the treatment of agitation in patients with dementia of the Alzheimer's Type.^68^ Otsuka Pharmaceutical is also conducting ongoing phase 3 trial to evaluate the efficacy, dose-response and safety of aripiprazole at 2, 3 and 6 mg per day in comparison with placebo in patients with agitation associated with AD.^69^ Meanwhile, Acadia Pharmaceuticals phase 2 study is evaluating the efficacy of pimavanserin compared with placebo following 12 weeks of treatment for agitation and aggression in AD.^70^

When completed, these ongoing studies will hopefully achieve a balance of efficacy over safety for drugs used in treating dementia-related agitation and other neuropsychiatric symptoms.

## Anecdote of clinical experience

Over the past three years, we have experience a surge in the number of dementia-related illnesses being admitted to our unit. Quite recently, we managed a clinical condition that apparently stimulates the urge for writing this review. The intention is to increase the awareness of fellow clinicians/physicians, and also, hoping the carers and family of dementia patients who read medical journals will be better informed.

An elderly frail woman, mostly bedbound, from a nursing home was admitted to our unit. She had background history of frontotemporal dementia and previous stroke. Her family mentioned that she often became agitated whenever she had exacerbations of arthritic pains or infections (particularly urinary infection). However, due to rising frequencies of agitation and aggression symptoms in the nursing home, she was reviewed by the community Psychiatry team and was started on quetiapine treatment. On subsequent review by the latter, risperidone was added to her medications due to lack of improvement of her symptoms. The addition of risperidone made her to be overly drowsy on most time of the day, and her oral intake became very poor according to her family.

Unfortunately, in just over six months on quetiapine and risperidone combination, she suffered another major ischemic stroke, and was started on end of life care in the same nursing home. At that time, all her active medications were stopped including the antipsychotics (risperidone and quetiapine). Surprisingly, she made a spontaneous recovery after few weeks, and her oral intake began to improve at the time. However, about two months following her recovery, she was restarted on her regular medications including the antipsychotics. Again, she started becoming drowsy again at most times of the day with worsening oral intake due to drowsiness. Little wonder, she presented to our unit with acute kidney injury from severe dehydration and also had aspiration pneumonia. During the week-long period of admission on our unit, her antipsychotics medications were stopped and she was rehydrated and treated for likely aspiration pneumonia. Her oral intake became significantly improved and she was neither agitated nor aggressive at any time during the period of her admission into our unit. On discharge, I discussed with the patient's next of kin the need to stop both the risperidone and quetiapine, as these medications were no longer indicated in the patient. I also put a note to the psychiatry team to inform them of the above plan and requested a follow-up by them.

From the above case, it is debatable whether or not the antipsychotic medications were the cause of her second stroke or perhaps, contributed to its occurrence. Also, the dehydration and likely aspiration pneumonia might arguably be connected to the excessive sedative effects of her antipsychotics medications. It is also unclear how much training the nursing home staffs had in caring for dementia patients generally, and how much of non-pharmacological approach was recommended by the psychiatry team before the start of the antipsychotic medications whose efficacy was questionable and with proven adverse effects?

Not surprisingly, van der Ploeg *et al.*^71^ in their research on 17 aged care facilities in Southeast Melbourne, Australia, report that non-pharmacological interventions are rarely implemented in aged care facilities despite their proven beneficial effects for agitated patients. They cited staff members’ lack of time as the main reason for the poor implementation of non-pharmacological interventions. Unfortunately, the above reason might just be applicable to hospitals as well, explaining why many clinicians have low threshold for commencing drug treatment at the slightest presentation of agitation by dementia patients, lacking the patience to embrace non-pharmacological approach as the initial first line treatment interventions. Some clinicians even commence dementia patients with previous history of agitation or other neuropsychiatric symptoms on longstanding antipsychotics and/or anxiolytics/hypnotics drug as maintenance treatment, ignoring several warnings by FDA and recommendations from many associations of geriatrics and psychiatry societies advising on careful use of drug interventions.

## Conclusion

As dementia is increasingly becoming more prevalent in this day and age, unfortunately, the treatment of dementia-related agitation and other behavioral symptoms have been a challenge. All the potential pharmacological treatment interventions present risks as well as concerns on their efficacy. Some pharmacotherapy of proven effectiveness, such as dextromethorphan-quinidine are not without safety concerns. Likewise, the non-pharmacological management which is the accepted first-line treatment approach may also not be effective in managing severe agitation and other behavioral symptoms in every dementia patient.

Future studies will be needed to focus on exploring the efficacy of non-pharmacological interventions and palliative care approach in advanced dementia, as well as determining the safety, tolerability and efficacy of the drugs treatment in managing the neuropsychiatric symptoms of dementia.

This review provides opportunity for increase knowledge and awareness of the non-pharmacological interventions among physicians, especially the clinicians with no specialty training in geriatrics medicine but, see dementia patients with agitation and other behavioral symptoms from time to time. Likewise, it hopefully will benefit all the readers of medical sciences to update their existing knowledge on matters relating to the management of dementia-related agitation.

## Figures and Tables

**Table 1:** Summary of studies on non-pharmacological interventions for managing agitation and behavioral symptoms of dementia

*Article*	*Participants/studies*	*Intervention(s)*	*Duration*	*Outcome*
Hawranik *et al.*^15^	51 participants	Therapeutic Touch (TT) versus Usual Care (UC).	2 weeks	After 5 days post interventions, both groups (TT and UC) showed improvements in both the physically aggressive behaviors (χ^2^=24.53, *P* <0.001) and physically nonaggressive behaviors (χ^2^=28.18, *P* <0.0001). However, both the physically aggressive and nonaggressive behaviors increased during the 2-week period after the completion of the interventions (χ^2^=10.63, *P* <0.01; incidence ratio=0.29, CI 0.13, 0.65 and χ^2^=11.03, *P* <0.01, respectively. Physically nonaggressive behaviors in the UC group were 2.3 times higher than in the TT group (CI.66, 7.81).
				
Woods *et al.*^16^	57 participants	Therapeutic Touch (TT) versus Usual Care (UC).	3 days	Statistically significant change was evident in the TT group when compared with the UC group, particularly in the frequency and intensity of behavioral symptoms, which demonstrated that TT offers a clinically more relevant decrease in behavioral symptoms of dementia. Both restlessness, *t*(36)=−2.435, *P*=0.020, and vocalization, *t*(36)=−2.261, *P* −0.030, were significantly improved in the TT group compared with the UC group.
				
Gitlin *et al.*^13^	60 participants (with 60 caregivers)	8 sessions of Tailored Activity Program (TAP) versus wait-list control	4 months period.	Outcome measures included behavioral occurrences, activity engagement, and quality of life in dementia patients together with the objective and subjective burden and skill enhancement in caregivers. Compared with controls, TAP intervention caregivers reported reduced frequency of odd behaviors (*P*=0.010; Cohen’s d=0.72), specifically for shadowing (*P*=0.003, Cohen’s d=3.10) and repetitive questioning (*P*=0.23, Cohen’s d=1.22); greater activity engagement (*P*=0.029, Cohen’s d=0.61. Fewer TAP intervention caregivers also reported agitation (*P*=0.014, Cohen’s d=0.75) or argumentation (*P*=0.010, Cohen’s d=0.77). Caregiver benefits included fewer hours doing things (*P* =0.005, Cohen’s d=1.14) and being on duty (*P*=0.001, Cohen’s d=1.01), greater mastery (*P*=0.013, Cohen’s d=0.55), self-efficacy (*P*=0.011, Cohen’s d=0.74).
				
O'Connor *et al.*^17^	64 participants	Dermally-applied, neuro-physiologically active, high purity 30% lavender oil versus inactive control (Jojoba) oil.	Three exposures over 1week period with a four-day washout period in-between.	First 30 min post-exposure: Mean (s.d.) for Lavender: Behavior −14.5 (10.8); Positive affect −7.0 (10.1); Negative affect 0.9 (3.9), and for Control: Behavior 16.0 (10.4); Positive affect 6.4 (9.9); Negative affect 1.0 (3.9). Second 30 min post-exposure: Mean (s.d.) for Lavender: Behavior 14.4 (10.6); Positive affect 6.7 (10.2); Negative affect 0.7 (3.9) and for Control: Behavior 15.5 (10.7); Positive affect 6.3 (9.6); Negative affect 0.9 (3.8) Lavender oil showed no significant beneficial effects over inactive control oil in treating agitation behaviors in dementia people. The decreased agitated behaviors noted in the lavender oil group were believed to be evident before exposure to the lavender.
				
Livingston *et al.*^18^	33 studies	Music therapy; sensory interventions; light therapy; communication skills training; dementia care mapping; aromatherapy; cognitive–behavioural therapy and stimulation therapy exercise	Varies with intervention (from 30 min and up to 6 months)	Comparison of interventions outcomes were estimated using standardised effect sizes (SES) with 95% confidence intervals. Training care-home staffs in communication skills, person-centred care and dementia care mapping with supervision during implementation were significantly effective for reducing severe agitation immediately (SES=0.3–1.8) and for up to 6 months afterwards (SES=0.2–2.2). Sensory intervention and music therapy also decreased overall agitation. Aromatherapy, exercise and light therapy failed to show any significant efficacy.
				
Abraha *et al.*^19, 20^	38 secondary studies (extracted from 142 primary studies).	Sensory stimulation interventions; cognitive/emotion-oriented interventions; and other therapies (exercise therapy, animal-assisted therapy)	Varies (up to 12 months)	Outcome measured were (1) multi-domain scales (Neuropsychiatric Inventory (NPI), Brief Psychiatric Rating Scale, BPRS), (2) scales specific to agitation (Cohen-Mansfield Agitation Inventory, CMAI) and (3) scales specific to depression or anxiety (Cornell Scale for Depression in Dementia, CSDD). Music therapy demonstrated efficacy in reducing agitation (SMD, −0.49; 95% CI −0.82 to −0.17; *P*=0.003), and anxiety (SMD, −0.64; 95% CI −1.05 to −0.24; *P*=0.002). Home-based behavioral management techniques, caregiver-based interventions or staff training in communication skills, person-centred care or dementia care mapping with supervision during implementation were found to be effective for symptomatic and severe agitation.
				
Vink *et al.*^21^	91 participants	Music therapy intervention compared with general day (recreational) activities	4 months	Both interventions resulted in short-term decrease in agitation. Although the music therapy reduced agitation behaviour much better, their difference was not statistically significant (F = 2.885, *P* = 0.090) and disappeared completely after adjustment for Global Deterioration Scale stage (F = 1.500; *P* = 0.222).
				
Jutkowitz *et al.*^22^	19 studies (3566 participants)	Dementia care mapping (DCM); Person-centered care (PCC); Clinical protocols to reduce the use of antipsychotic and other psychotropic drugs; and Emotion-oriented care.	Varies (2 weeks to 20 months)	DCM (standardized mean difference −0.12, 95% confidence interval (CI)=−0.66 to 0.42), PCC (standardized mean difference −0.15, 95% CI=−0.67 to 0.38), and Protocols to reduce antipsychotic and other psychotropic use (Cohen-Mansfield Agitation Inventory mean difference −4.5, 95% C=−38.84 to 29.93). Insufficient strength of evidence to conclude that behavioral management techniques or interventions are any more effective than the usual care in improving agitation and aggression in dementia populations.
				
Acharya *et al.*^9^	23 participants	Electroconvulsive therapy (ECT)		Outcome measures included Cohen-Mansfield Agitation Inventory (CMAI)-short form, Neuropsychiatric Inventory (NPI)-Nursing Home Version, Cornell Scale for Depression in Dementia (CSDD), and the Clinical Global Impression Scale (CGI) at baseline, during, and after ECT sessions, and within 72 h before discharge. Regression analyses revealed a significant decrease from baseline to discharge on the CMAI (F (4, 8)=13.3; *P*=0.006) and NPI (F(4, 31)=14.6; *P*<0.001). There was no statistically significant change in scores on the CSDD. The CGI scores on average changed from a rating of ‘markedly agitated/aggressive’ at baseline to ‘borderline agitated/aggressive’ at discharge.
				
Yang *et al.*^11^	186 participants	Aroma-acupressure (A-a) and Aromatherapy (A) interventions versus Control group.	4 weeks	The differences in agitation were assessed using Cohen-Mansfield Agitation Inventory (CMAI) scale and the Heart Rate Variability (HRV) index. The CMAI scores were significantly lower in the aroma-acupressure and aromatherapy groups compared with the control group in the post-test and post-three-week assessments On the basis of the HRV and the CMAI, aroma-acupressure showed better improvement at reducing agitation, inhibit the sympathetic nervous system, and activate the parasympathetic nervous system compared with aromatherapy.
				
				*Group* *Pre-test mean±s.d.* *Post-test mean* *Post 3 weeks* *β (95% CI)* P *value*
				A-a group (*n*=56) 54.58±11.01 43.24±10.00 51.21±11.95 16.74 (13.71−19.77) 0.00*
				A group (*n*=73) 41.81±7.89 41.08±8.24 39.80±7.27 4.01 (1.19−6.83) 0.01*
				Control group (*n*=57) 37.68±4.12 41.72±5.08 42.13±5.53 Reference —
				Time — — — — —
				Post-test — — — 3.96 (2.22−5.71) <0.01
				Post-3-week — — — 4.39 (2.64−6.13) <0.01

**Table 2:** Summary of studies on pharmacological interventions for managing agitation and behavioral symptoms of dementia

*Article*	*Participants/total studies*	*Intervention(s)*	*Duration*	*Outcome*
Ballard *et al.*^5^	165 participants	Continue neuroleptic treatment for 12 months or a switch to placebo.	12 months	Neuropsychiatric symptoms evaluated with the Neuropsychiatric Inventory (NPI). At 6 months: No significant difference between the continue treatment and placebo groups. Estimated mean change in Severe Impairment Battery (SIB) scores between baseline and 6 months showed estimated mean difference in deterioration (favouring placebo) −0.4 (95% confidence interval [CI] −6.4 to 5.5), adjusted for baseline value (*P*=0.9). For neuropsychiatric symptoms, the estimated mean difference in deterioration (favouring continued treatment) was 2.4 (95% CI −8.2 to 3.5), adjusted for baseline value (*P*=0.4). At 12 months: clinically important but no statistically significant difference between the continue treatment and placebo groups in the estimated mean change in SIB scores between baseline and 12 months. Estimated mean difference in deterioration (favouring placebo) 8.4 (95% CI −18.6 to 1.7), adjusted for baseline value (*P* ¼ 0.1). For the NPI, there was a significant difference between the continue treatment and placebo groups in the estimated mean change in NPI scores between baseline and 12 months, with 11.4 points (s.d. 17.7) deterioration for the placebo group compared with a 1.4 (s.d. 22.1) deterioration for the continue treatment group. Estimated mean difference in deterioration (favouring continued treatment) 10.9 (95% CI 20.1–1.7), adjusted for baseline value (*P* ¼ 0.02).
Rappaport *et al.*^23^	129 participants	IM aripiprazole (5, 10, or 15 mg) versus IM placebo.	24 h	Efficacy analyses done included the Positive and Negative Syndrome Scale-Excited Component (PEC) scores, Agitation–Calmness Evaluation Scale (ACES), Clinical Global Impressions–Severity of Illness (CGI-S) and Clinical Global Impressions–Improvement (CGI-I) rating scales. PEC scores showed greater improvements in agitation with IM aripiprazole 10 mg and 15 mg compared with IM placebo. Mean CGI-I score was lower for all 3 IM aripiprazole dose groups versus IM placebo. Generally, there were significant improvements on the PEC, CGI-I, CGI-S and ACES rating scales with IM aripiprazole compared with IM placebo Likewise, more adverse effects (AEs) were reported with IM aripiprazole (50% to 60%) than IM placebo (32.0%), but over 90% were mild or moderate in severity.
Cakir and Kulaksizoglu^24^	16 participants	Mirtazapine (15–30 mg)	12 weeks	Changes in Cohen-Mansfield Agitation Inventory-Short form (CMAI-SF) scores and Impression-Severity scale (CGI-S) scores with tolerability-safety profile were the primary and secondary outcome measurements respectively. Statistically significant improvement between baseline and 12th week in both CGI-S and CMAI-SF scores (*P*<0.001). The mean change in CMAI-SF was −11.0 (s.d.±7.5) and −2.0 (s.d.±0.9) in CGI-S, *P* value was<0.001 in both. Mirtazapine demonstrated some efficacy in treating agitated patients with Alzheimer's disease. Side-effect profile of mirtazapine reported includes sedation, headache, increased appetite (but no weight gain) and mild hypotension.
Wang *et al.*^25^	22 participants	Prazocin versus Placebo	8 weeks	Brief Psychiatric Rating Scale (BPRS) and Neuropsychiatric Inventory (NPI) at Weeks 1, 2, 4, 6 and 8; and the Clinical Global Impression of Change (CGIC) at Week 8 were measured. Participants that had prazosin (mean dose: 5.7±0.9 mg/day) show more improvements than those that had placebo (mean dose: 5.6±1.2 mg/day) on the NPI (mean change: −19±21 versus -2±15, chi=6.32, df=1, *P*=0.012) and BPRS (mean change: -9±9 versus -3±5, chi=4.42, df=1, *P*=0.036) based on linear mixed effects models and the CGIC (mean: 2.6±1.0 versus 4.5±1.6, z=2.57, *P*=0.011 [Mann–Whitney test]). The AEs reported by both prazosin and placebo groups were similar.
Lockhart *et al.*^26^	6 studies (1393 participants)	Donepezil and memantine monotherapy versus Placebo in managing the behavioral and psychological symptoms of dementia (BPSD)	24 weeks	Absolute change in NPI score were compared with baseline NPI score. Results from the total NPI measured showed that BPSD demonstrated statistically significant improvement with donepezil monotherapy within its licensed indication compared with placebo [weighted mean difference (WMD) in NPI -3.51, 95% confidence interval (CI) −5.75, −1.27]. Contrarily, there was no statistically significant difference between memantine monotherapy used within its license compared with placebo (WMD −1.65, 95% CI −4.78, 1.49). Also, WMD in NPI for donepezil versus memantine favored donepezil but this was not statistically significant (−1.86, 95% CI −5.71, 1.99; *P*=0.34).
Fox *et al.*^27^	153 participants	Memantine versus Placebo.	12 weeks	Primary outcome was 6 weeks Cohen-Mansfield Agitation Inventory (CMAI) and Secondary outcomes included 12 weeks CMAI; 6 and 12 weeks Neuropsychiatric symptoms (NPI), Clinical Global Impression Change (CGI-C), Standardised Mini Mental State Examination, Severe Impairment Battery. No significant difference in the primary outcome, 6 weeks CMAI between memantine and placebo (memantine lower −3.0; −8.3 to 2.2, *P* = 0.26); or 12 weeks CMAI; or CGI-C or adverse events at 6 or 12 weeks. NPI mean difference favored memantine at weeks 6 (−6.9; −12.2 to −1.6; *P* = 0.012) and 12 (−9.6; −15.0 to −4.3 *P* = 0.0005). Memantine was significantly better than placebo for cognition, but failed to show any significant benefits over placebo at improving agitation in AD people.
Herrmann *et al.*^28^	369 participants	Efficacy of Memantine versus Placebo.	24 weeks	Total NPI and Severe Impairment Battery (SIB) scores for behavior and cognition respectively, were the primary outcome measured. Other endpoints included the Clinician's Interview-Based Impression of Change Plus Caregiver Input (CIBIC-Plus) and the Cohen-Mansfield Agitation Inventory (CMAI) total score. There were no statistically significant differences between memantine and placebo in mean change from baseline in NPI (*P*=0.42), SIB (*P*=0.60), or in any of the secondary outcome measured. Behavior improved in both groups (total NPI change scores -3.90±1.24 for memantine and −5.13±1.23 for placebo).
Li *et al.*^29^	42 participants	Efficacy of 10 mg memantine on neuropsychiatric symptoms of patients with mild and moderate-to-severe form of behavioral variant fronto-temporal dementia (bvFTD).	6 months	Primary and secondary endpoints included Neuropsychiatric Inventory Questionnaire (NPI-Q); Clinic Dementia Rating (CDR) scores; Inventory Caregiver Distress Scale (NPI-D), MMSE, Montreal Cognitive Assessment (MoCA) and Hamilton Depression Rating Scale (HAMD) scores. In both baseline and final visit, no statistically significant differences were detected in the NPI-D and HAMD scores. However, following 6 months of memantine treatment, the subgroup of patients with moderate-to-severe bvFTD exhibited significantly improved total NPI-Q scores (Z=−2.488, *P*=0.013), and improvements in the subscales of agitation (Z=−2.058, *P*=0.04) compared with those at baseline. By contrast, memantine caused no significant changes in patients with mild bvFTD with regard to the total NPI-Q score (14.50±2.82 baseline versus 15.70±3.00 after 6 months, *P*=0.192) or individual subscale scores.
Trzepacz *et al.*^30^	132 participants	Mibampator versus Placebo	12 weeks	Primary endpoints included 4-domain A/A subscale of the Neuropsychiatric Inventory(NPI-4- A/A), and Secondary measures were Cohen-Mansfield Agitation Inventory, Cornell Scale for Depression in Dementia, Frontal Systems Behavior Inventory (FrSBe), and Alzheimer's Disease Assessment Scale-Cognitive. Both mibampator and placebo groups improved on the NPI-4- A/A. However, among secondary endpoints measured, mibampator was significantly better (*P*=.007) than placebo only on the FrSBe. Both groups had similar adverse effects.
Porsteinsson *et al.*^31^	186 participants	Citalopram versus Placebo.	9 weeks	18-point Neurobehavioral Rating Scale agitation subscale (NBRS-A), modified Alzheimer Disease Cooperative Study-Clinical Global Impression of Change (mADCS-CGIC) and Cohen-Mansfield Agitation Inventory (CMAI), and Neuropsychiatric Inventory (NPI), were among the outcome measured. Citalopram showed significant improvement compared with placebo on all outcomes measured including the total NPI, except on the NPI agitation subscale. The NBRS-A estimated treatment difference at week 9 (citalopram minus placebo) was −0.93 (95% CI, −1.80 to −0.06), *P* = 0.04. Also, mADCS-CGIC showed 40% of citalopram participants having significant improvement from baseline compared with 26% of placebo recipients, with estimated treatment effect (odds ratio [OR] of being at or better than a given CGIC category) of 2.13 (95% CI, 1.23-3.69), *P* = 0.01. Worsening of cognition (−1.05 points; 95% CI, −1.97 to −0.13; *P* = 0.03) and QT interval prolongation (18.1 ms; 95% CI, 6.1-30.1; *P* = 0.01) were seen in the citalopram group.
Cummings *et al.*^32^	220 participants	Combination of dextromethorphan-quinidine treatment versus placebo.	10 weeks	The efficacy endpoints were changes from baseline in the NPI total scores. Analysis combining all participants showed significantly reduced NPI Agitation/Aggression scores for dextromethorphan-quinidine compared with placebo (ordinary least squares z statistic, −3.95; *P* < .001). Most commonly reported AEs (>3% and greater than placebo) were falls (8.6% versus 3.9%), diarrhea (5.9% versus 3.1%), urinary tract infection (5.3% versus 3.9%) and dizziness (4.6% versus 2.4%) for dextromethorphan-quinidine versus placebo respectively.
